# Absinthe

**Published:** 1889-12-07

**Authors:** 


### ABSINTHE.

That indulgence in this spirit, so fearfully prevalent in France, is more hurtful than that in Eau de Vie, or even gin, has long been acknowledged, and Dr. Magnan, in a report published in 1874, maintained that the effects were not merely those of alcoholism, but of that plus absinthism, absinthe alone being capable of inducing epileptiform convulsions. More recently, the adulteration of spirits with methyl salicylate, salicylic aldehyde, etc., has been urged as an aggravation, and, lastly, MM. Cadeac and Meunier have attempted to vindicate the reputation of absinthe, which they allege to be a harmless and, indeed, a wholesome tonic, transferring the blame to its adulterations, the essences of anise, star anise, and fennel, especially the first, and proposing to substitute the expression anisism for absinthism. But M. Laborde has shown that the true absinthe does produce the nervous symptoms in question, though anise, etc., may do so likewise, and that MM. Cadeac and Meunier had experimented with the so-called Algerian absinthe, distilled from the root of the asphodel, a cheap adulteration, which from its harmless character deserves to be more used.

